# The Effect of Smoking on the Immune Microenvironment and Immunogenicity and Its Relationship With the Prognosis of Immune Checkpoint Inhibitors in Non-small Cell Lung Cancer

**DOI:** 10.3389/fcell.2021.745859

**Published:** 2021-09-28

**Authors:** Yueqin Sun, Qi Yang, Jie Shen, Ting Wei, Weitao Shen, Nan Zhang, Peng Luo, Jian Zhang

**Affiliations:** Department of Oncology, Zhujiang Hospital, Southern Medical University, Guangzhou, China

**Keywords:** non-small cell lung cancer, immune checkpoint inhibitors, prognosis, bioinformatics, mass cytometry

## Abstract

**Background:** The emergence of immune checkpoint inhibitors (ICIs) has opened a new chapter for the treatment of non-small cell lung cancer (NSCLC), and the best beneficiaries of ICI treatment are still being explored. Smoking status has been repeatedly confirmed to affect the efficacy of ICIs in NSCLC patients, but the specific mechanism is still unclear.

**Methods:** We performed analysis on the Memorial Sloan Kettering Cancer Center (MSKCC) clinical NSCLC cohort receiving ICI treatment, The Cancer Genome Atlas (TCGA) Pan-Lung Cancer cohort, and Gene Expression Omnibus (GEO) database GSE41271 lung cancer cohort that did not receive ICI treatment, including survival prognosis, gene mutation, copy number variation, immunogenicity, and immune microenvironment, and explored the impact of smoking status on the prognosis of NSCLC patients treated with ICIs and possible mechanism. In addition, 8 fresh NSCLC surgical tissue samples were collected for mass cytometry (CyTOF) experiments to further characterize the immune characteristics and verify the mechanism.

**Result:** Through the analysis of the clinical data of the NSCLC cohort treated with ICIs in MSKCC, it was found that the smokers in NSCLC receiving ICI treatment had a longer progression-free survival (HR: 0.69, 95% CI: 0.49–0.97, *p* = 0.031) than those who never smoked. Further analysis of the TCGA and GEO validation cohorts found that the differences in prognosis between different groups may be related to the smoking group’s higher immunogenicity, higher gene mutations, and stronger immune microenvironment. The results of the CyTOF experiment further found that the immune microenvironment of smoking group was characterized by higher expression of immune positive regulatory chemokine, and higher abundance of immune activated cells, including follicular helper CD4^+^ T cells, gamma delta CD4^+^ T cells, activated DC, and activated CD8^+^ T cells. In contrast, the immune microenvironment of non-smoking group was significantly enriched for immunosuppressive related cells, including regulatory T cells and M2 macrophages. Finally, we also found highly enriched CD45RA^high^CD4^+^ T cells and CD45RA^high^CD8^+^ T cells in the non-smoking group.

**Conclusion:** Our research results suggest that among NSCLC patients receiving ICI treatment, the stronger immunogenicity and activated immune microenvironment of the smoking group make their prognosis better.

## Introduction

Lung cancer is one of the most frequently occurring types of tumor malignancy with the highest incidence worldwide. However, the 60-month overall survival rate of patients remains extremely poor and is less than 10% of stage IV patients. Smoking is one of the leading known risk factors (Herbst et al., 2018; Duma et al., 2019). Immune checkpoint inhibitors (ICIs) are mainly used to treat programmed cell death 1 (PD-1), programmed cell death ligand 1 (PD-L1), and cytotoxic T lymphocyte antigen 4 (CTLA4) pathways. In recent years, ICIs have achieved significant therapeutic effects in the treatment of advanced NSCLC. Multiple monoclonal anti-PD-1/PD-L1 monoclonal antibodies (mAbs) (including nivolumab, pembrolizumab, and atezolizumab) have been approved by the U.S. Food and Drug Administration to treat advanced NSCLC, further confirming the importance of ICIs in the treatment of advanced NSCLC (Constantinidou et al., 2019; Lin et al., 2019).

However, the ICIs’ therapeutic effect in NSCLC is affected by many factors: a healthy intestinal microbial environment, antibiotic use, and smoking status all affect its efficacy (Yi et al., 2018; Pinato et al., 2019). At present, the PD-L1 expression level, tumor mutation burden (TMB), microsatellite instability (MSI), and DNA mismatch repair (dMMR) are also used as indicators to evaluate the efficacy of ICIs in treating NSCLC patients. However, due to individual differences and other factors, all these indicators have specific limitations. Therefore, determining the best beneficiaries is crucial for developing clinical NSCLC treatment strategies (Kim et al., 2019; Ruiz-Banobre and Goel, 2019; Chiu et al., 2020). Several study cohorts have shown the impact of different smoking statuses specific to the treatment of ICIs. Gainor et al.’s (2020) retrospective analysis showed that different smoking frequencies could lead to different immunobiological characteristics in patients, influencing the efficacy of treatment with ICIs. Patients who are heavy smokers have better PFS (progression-free survival) and DOR (duration of response) in the treatment of PD-1 (Gainor et al., 2020). Several meta-analyses also show that previous or current smokers are more likely to benefit from treatment with ICIs than non-smokers (El-Osta and Jafri, 2019; Raphael et al., 2020).

Although current studies are inclined toward the analysis of smoking status and the efficacy of treatment with ICIs and prognosis, there has been little exploration of its specific mechanisms. Nevertheless, most literature considers the differences in the immune microenvironment among different groups. Likewise, Li et al. (2018) and Sui et al. (2020) analyzed the microenvironmental differences between smokers and non-smokers with lung adenocarcinomas, and found relatively high proportions of CD8^+^ T cells, activated CD4^+^ T cells, and M1 macrophages in smokers with lung adenocarcinomas (Kinoshita et al., 2016). At the same time, Pan et al. (2017) also demonstrated higher PD-L1 expression in patients who are smokers, which is directly related to the efficacy of ICIs, so it has also been widely explored as a possible factor. However, the hypothesis regarding this mechanism is relatively one-sided and unsubstantiated. Both only analyze the slight differences between the immune infiltrating cells between a certain histological tumor type or the omics data of mRNA. There is currently no literature on the overall immune microenvironment and immunogenicity of smoking and non-smoking NSCLC patients at the multi-omics and single-cell levels.

In our study, we attempt to further understand the association between the treatment of NSCLC patients with ICIs and these patients’ history of smoking. Thus, we first analyzed the genomic differences between previous or current smokers and non-smokers from multiple databases such as TCGA, MSKCC, GEO (including TMB, mutation status, copy number variation (CNV), immunogenicity, immune-related gene expression profiles (GEPs), and signaling pathways). We then used samples from NSCLC patients to verify the results with CyTOF experiments. We aim to explore the overall differences in the multi-omics, immune microenvironment, and the mechanism of prognostic differences between the history of smoking of NSCLC patients treated with ICIs and provide clear guidance for the clinical selection of patients suited to treatment with ICIs.

## Materials and Methods

### Data Source

To assess the effect of smoking history on the efficacy of treating NSCLC patients with ICIs, from cBioportal (Cerami et al., 2012; Gao et al., 2013) we downloaded the NSCLC cohort from MSKCC with a history of smoking and treatment with ICIs as the discovery cohort^1^ [reported by Rizvi et al. (2018)], along with the analysis of survival, immunogenicity, and gene mutation, for a total of 240 cases. At the same time, we used cohorts from TCGA Pan-Lung Cancer^2^ (Campbell et al., 2016) on cBioPortal and the NSCLC cohort, GEO:GSE41271, the largest number of cases in the history of smoking, from the GEO database as verification to evaluate and verify the differences in genomics between previous or current smokers, and non-smokers with NSCLC. After removing patients whose smoking history had not been recorded, there remained 1,087 cases in the TCGA Pan-Lung Cancer cohort and 271 cases in the GSE41271 cohort. According to smoking history, each cohort was divided into a previous or current smoking group, and a non-smoking group for the comparative analysis.

### Survival Analysis

The R package “survival” and “survminer” were used to calculate the survival analysis outcome indicators: PFS (progression-free survival) and OS (overall survival). The same software and packages were used to perform the survival analysis and visualization of the survival curve of the MSKCC cohort and the TCGA Pan-Lung Cancer cohort.

### Immunogenicity Analysis

Consistent with other literature, the somatic mutation data in 240 NSCLC samples reported by Rizvi et al. were obtained from targeted next-generation sequencing (NGS; MSK-IMPACT) (Chalmers et al., 2017). The non-synonymous mutations in the immunotherapy cohort (Rizvi et al.) were used as the raw mutation count and divided by 38 Mb to quantify the tumor mutation burden (TMB). In 87% of TCGA Pan-Lung Cancer cases (942 cases), TMB, indel, and SNV neoantigen load data have been reported in the relevant literature (Thorsson et al., 2018). The formula *log(1* + *TMB)* was used to normalize the TMB results. The R package “ggplot2” (Wilkinson, 2011) was used to visualize the results of the TMB and neoantigen load difference analysis. Unless otherwise specified, ggplot2 was used throughout this article to visualize the results of analyses.

### Gene Mutation and Copy Number Variation Analysis

The gene mutation data of the Rizvi et al. cohort was downloaded directly from cBioportal. Due to the lack of cBioportal mutation data in the TCGA Pan-Lung Cancer cohort, we used the TCGA Barcode as our target and used the R package “TCGAbiolinks” (Colaprico et al., 2016) to download the corresponding mutation maf file from the official TCGA website^3^, and included 952 cases with mutation data and smoking history records. The R package “complexHeatmap” (Gu et al., 2016) was used to visualize the top 20 gene mutations and corresponding clinical features in the immunotherapy cohort in the MSKCC and the TCGA Pan-Lung Cancer cohort. For MSKCC queue and TCGA Pan-Lung Cancer queue, CNV segments (hg19) were downloaded from cBioportal and analyzed using GenePattern (Hubble et al., 2009)^4^ GISTIC 2.0. We used the R package “Maftools” (Mayakonda et al., 2018) to visualize the CNV of the results of the GISTIC2.0 analysis.

### Immune Characteristic Analysis

CIBERSORT (Newman et al., 2015)^5^ was used to analyze the gene expression data (Illumina HiSeq, RNA-Seq) of the TCGA Pan-Lung Cancer cohort downloaded from TCGAbiolinks. The gene expression data of the GSE41271 cohort (Illumina HumanWG-6 v3.0 expression beadchip) was downloaded from GEO to compare the infiltration state of 22 immune cells in the smoking group and the non-smoking group. In addition, we used the R package “edgeR” (Robinson et al., 2010) to compare the mRNA expression of immune-related genes in the smoking group and non-smoking group in the TCGA Pan-Lung Cancer cohort, and use the package “limma” (Law et al., 2016) to compare the same groups and variables in the GSE41271 cohort.

### Gene Difference Analysis and Enrichment Pathway Analysis

The R package “edgeR” was used to analyze the difference in gene expression data (raw count) in the TCGA Pan-Lung Cancer cohort, and we used the “limma” package to perform the genetic difference analysis of the GSE41271 cohort. The R package “clusterProfiler” (Yu et al., 2012) was used to reform a Gene Set Enrichment Analysis (GSEA) on the genes that were significantly different between the two cohorts. Among them, *p* < 0.05 in Gene Ontology (GO) terms, Kyoto Encyclopedia of Genes and Genomes (KEGG), and Reactome were considered to have significant differences.

### Mass Cytometry (CyTOF)

#### Specimen Source

Eight cases of tumor tissues of NSCLC patients undergoing surgical treatment were collected from the Department of Thoracic Surgery at the Zhujiang Hospital of the Southern Medical University. Each sample was about 1 cm^3^ in size. All specimens were approved and signed authorization for their use was obtained from the patients. The ethics committee of Zhujiang Hospital of Southern Medical University (Guangzhou, China) approved the specimen collection process.

#### Mass Cell Data Collection

After washing with RPMI 1640 medium, the fresh lung tumor samples were dissociated into single cells under the irradiation of deoxyribonuclease and type IV collagenase. ACK lysis buffer (PLT) was used to remove the red blood cells, and the number of live and dead cells was then counted to estimate the sampling efficiency. Cell-ID cisplatin 194Pt (Fluidigm) was used to identify the dead cells, after which block qualified samples were placed on ice for 20 min. Each sample was then incubated on ice for 30 min, with the surface antibody mixture (Maxpar Antibody Labeling Kit; Fluidigm) and without removing the blocking solution, using Maxpar Fix and Perm Buffer. The final 500 μMNA intercalator (Cell-ID Intercalator-Ir; Fluidigm) was incubated with 200 μl after the resuspended cells were washed in each sample and finally stored overnight at 4°C. Subsequently, intracellular staining was performed, the cells were washed with the intracellular antibody mixture on ice, pre-fixed, and co-incubated for 30 min. Then the cells were rinsed and then collected on the CyTOF system (Helios; Fluidigm) to detect the signal (Han et al., 2018). Antibody selection is shown in Supplementary Table 1.

#### Mass Cytometry Data Analysis

We sorted CD45^+^ cells, and used the FlowJo software and R package “cytofworkflow” (Nowicka et al., 2017) to complete quality control, clustering, cell annotation, and visualization. Then arcsinh with a cofactor of 5 was used when generating the SingleCellExperiment object. In addition, the cells were overclustered first (SOM = 100, maxK = 30), based on the expression of cell-specific markers, and then the same type of cells was re-clustered. If a cluster of cells highly expressed two different cell-specific markers (such as CD19 and CD3), they were defined as “Mixed_cell” and discarded before proceeding with the following analysis. The difference analysis of the subpopulation abundance between different cell types is done by the “diffcyt()” function of the diffcyt package in the cytofworkflow package, where gender and age are random variables, and the selected method is a generalized linear mixed model (GLMM). Benjamini–Hochberg was also used to correct the *p* value. When the *p* value is less than 0.05 and the false discovery rate is less than 0.05, the corresponding cell type is considered to be significantly different between smokers and non-smokers.

### Statistical Analysis

The Wilcoxon rank-sum test was used to compare the differences in TMB, indel/SNV neoantigen load, immune cell abundance, and immune-related gene expression between previous or current smokers, and the non-smokers. In addition, Fisher’s exact test was used to compare the difference between the smoking and non-smoking groups of the top 20 gene mutations in the immunotherapy cohort in MSKCC and the TCGA Pan-Lung Cancer cohort. The Kaplan–Meier method and log-rank two-sided tests were used for survival analysis, with *p* < 0.05 accepted as statistically significant. All statistical tests and visualization were done using the software R (version 4.0.3).

## Results

### Data Collection and Clinical Features

To study the difference between previous or current smokers, and non-smokers with NSCLC, we selected four cohorts for analysis. Three of the cohorts were from public databases, including Rizvi et al.’s discovery cohort after ICI treatment from the MSKCC database, the Pan-Lung Cancer cohort from the TCGA database, and NSCLCs in the GSE41271 dataset for non-ICI treated patient data from the GEO database; these data were also based on Wistuba II’s sequencing and analysis of 275 lung cancer specimens collected from MD Anderson Cancer Center between 1997 and 2005, which mainly includes adenocarcinomas (*n* = 183) and squamous carcinomas (*n* = 80).

**FIGURE 1:**
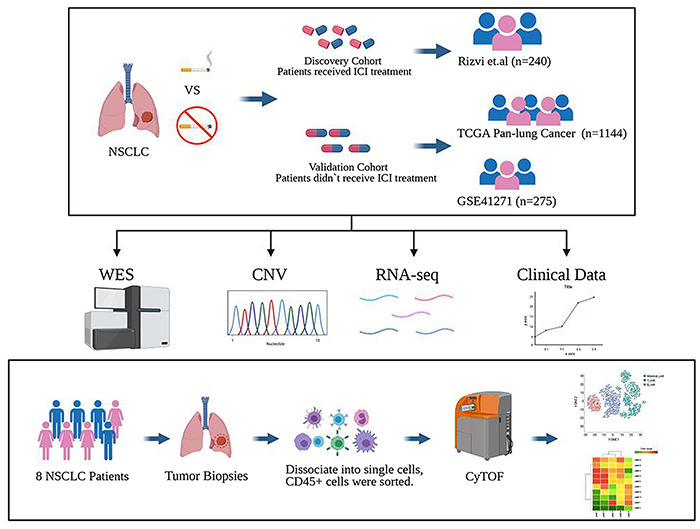
Study design. The discovery cohort from the MSKCC database included 240 NSCLC patients treated with ICIs. The validated cohorts from the TCGA database and GEO database (GSE41271) that were not treated with ICIs contained 1,144 patients and 275 patients. Also, a cohort of 8 cases of NSCLC who did not receive ICI treatment collected from Zhujiang Hospital of Southern Medical University underwent CyTOF experimental verification. Figure created with BioRender.com.

In the last cohort, we collected eight surgical samples of clinical NSCLC patients from Zhujiang Hospital of Southern Medical University for CyTOF analysis. These patients also had not received treatment with ICIs. Ultimately, a total of 1,667 patients were involved in all cohorts. After further excluding samples for which the patients’ history of smoking had not been recorded and non-NSCLC cases, a total of 1,606 patients were finally included for analysis. The flowchart is shown in Figure 1. The clinical characteristics of MSKCC data discovery cohort and the TCGA and GEO cohorts are summarized in Supplementary Tables 2–4, while those of the CyTOF cohort are summarized in Supplementary Table 5.

### Association of Smoking Status, Treatment With Immune Checkpoint Inhibitors, Prognosis, and Immunogenicity

**FIGURE 2:**
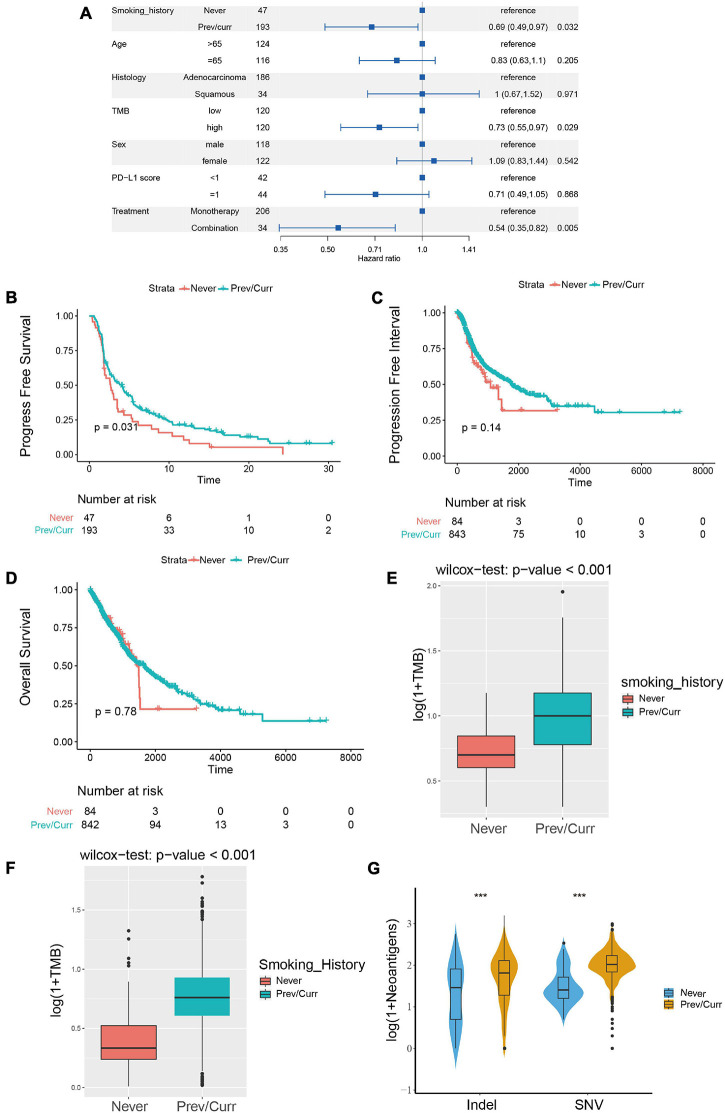
Association between the smoking status and clinical outcomes and immunogenicity in NSCLC. **(A)** Forest plots for the results of the univariate Cox regression analyses. The *p*-value of smoking status is less than 0.05, the main portion of the forest plot presents the hazard ratio (HR) and 95% CI, and the HR indicates the predictors of favorable (HR < 1) or poor (HR > 1) PFS. **(B)** Kaplan–Meier analysis was used to compare progression-free survival (PFS) of previous or current smokers with that of non-smokers in the ICI treatment in NSCLC cohort from MSKCC. **(C,D)** Kaplan–Meier analysis was used to compare progression-free interval (PFI) **(C)** and overall survival (OS) **(D)** of previous or current smokers with that of non-smokers in the TCGA Pan-Lung Cancer cohort (without ICI treatment). **(E)** Comparison of tumor mutational burden (TMB) between the previous or current smokers and non-smokers in the ICI treatment cohort from MSKCC. **(F)** Comparison of tumor mutational burden (TMB) between the previous or current smokers and non-smokers in the TCGA Pan-Lung Cancer. **(G)** Comparison of neoantigen load between the previous or current smokers and non-smokers in the TCGA Pan-Lung Cancer. **(E–G)** All expression values are logarithmized by log(1 + x) using Wilcoxon test for variance analysis. *p < 0.05; **p < 0.01; ***p < 0.001; ****p < 0.0001; ns, not significant.

To discover a suitable NSCLC population for treatment with ICIs, we performed a univariate Cox regression on the survival prognosis of the MSKCC data for the cohort treated with ICIs (Figure 2A). We found that smoking status, tumor mutation burden, and treatment options are significantly related to the prognosis of patients treated with ICIs. Furthermore, being a previous or current smoker, high TMB, and two-drug combination therapy with ICIs were associated with longer PFS (*p* < 0.05). The relationship between the TMB level, ICI combination, and the efficacy of ICIs has been confirmed in the relevant literature (Kim et al., 2019; Xu et al., 2020; Carretero-Gonzalez et al., 2021). However, there are few articles on smoking status and the prognosis of patients treated with ICIs. Therefore, our study further examines the relationship between this factor and the prognosis of NSCLC patients treated with ICIs; the survival curve shows longer PFS in past and current smokers than in non-smokers (Figure 2B). We also conducted a survival analysis of previous or current smokers compared with non-smokers in the TCGA cohort who were not treated with ICIs, and we found no significant difference in either PFI or OS (Figures 2C,D), which further shows that smoking status is only related to the prognosis of patients treated with ICIs.

Subsequently, we further analyzed the difference in TMB between the smoking and non-smoking groups in the ICI cohort and found that the TMB of the smoking group was significantly higher than that of the non-smoking group (Figure 2E, *p* < 0.001). We obtained the same result for the patients in the TCGA database; the TMB and neoantigen scores in the smoking group were significantly higher than those in the non-smoking group (Figures 2F,G). These results indicate that among the NSCLC patients treated with ICIs, the smoking group has a higher likelihood of receiving a preferable prognosis. Smoking status may affect the treatment of patients with ICIs via the difference in TMB and immunogenicity.

### Mutation Landscape and Copy Number Variation in Different Smoking Status

**FIGURE 3:**
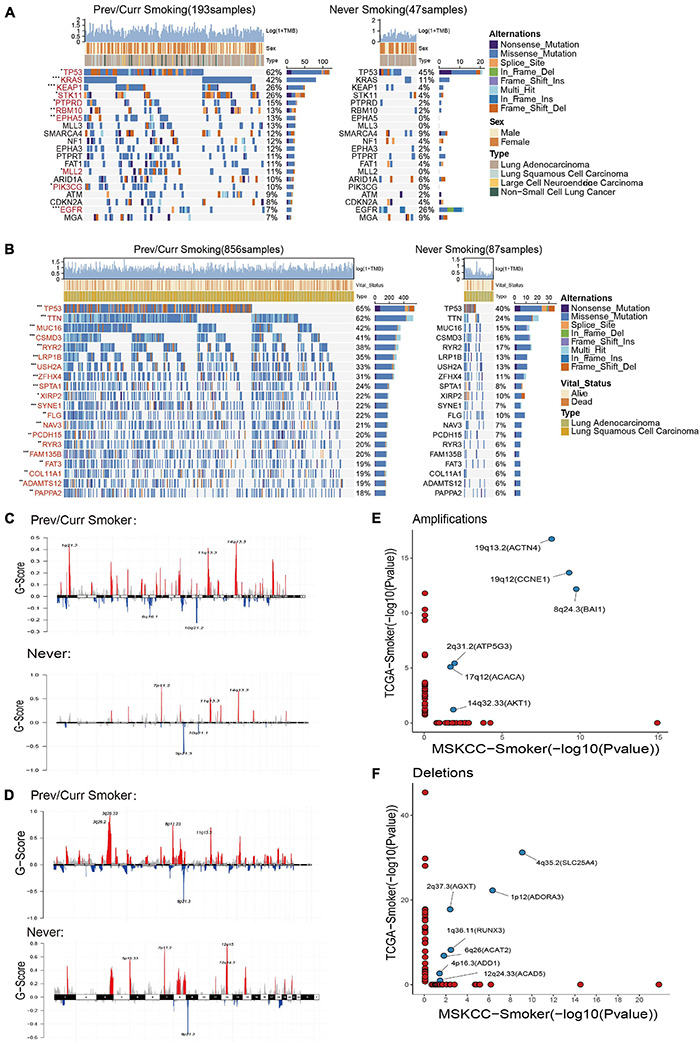
Mutation landscape and copy number variation in different smoking status. **(A)** Top 20 significantly mutated genes in the immunotherapy cohort of MSKCC database. The mutation landscape was divided into two groups according to smoking status, and genes were ranked by mutation frequencies. The sample type, sex, and TMB score are annotated in order in the top panel. The genes marked in red indicate that their mutation frequency is significantly different between the two groups in Fisher’s exact test (*p* < 0.05). **(B)** Top 20 significantly mutated genes in TCGA Pan-Lung Cancer. The mutation panorama was divided into two groups according to smoking status, and genes were ranked by mutation frequencies. The sample type, vital status, and TMB score are annotated in order in the top panel. The genes marked in red indicate that their mutation frequency is significantly different between the two groups in Fisher’s exact test (*p* < 0.05). **(C,D)** Maftools was used to visualize the copy number alteration (CNV) analysis based on GISTIC2.0 of the MSKCC cohort **(C)** and TCGA Pan-Lung Cancer cohort **(D)** under different smoking status. **(E,F)** The lollipop graph shows the significantly amplified **(E)** or deleted **(F)** sites in the smoking group in the MSKCC discovery cohort and the TCGA verification cohort and the main genes encoded by the chromosomal sites. The marked site in the middle is the intersection of the two databases. **(A,B)** *p < 0.05; **p < 0.01; ***p < 0.001; ****p < 0.0001; ns, not significant.

To further analyze the reasons leading to aforementioned results, we separately included the discovery cohort and the verification discovery cohort to analyze the difference in the mutation and CNV among the different groups. The MSKCC and the TCGA data mutation landscapes showed that the smoking group had a higher frequency of gene mutations than the non-smoking group (Figures 3A,B). The MSKCC data were based on the top 20 mutations in the total data; 90% of the gene mutation frequencies are higher in the smoking group than in the non-smoking group (Figure 3A). Fisher’s exact test indicated that TP53, KRAS, KEAP1, and other genes were significantly mutated in the smoking group (*p* < 0.05), and that EGFR is the only gene that is mutated significantly in the non-smoking group. Among them, TP53 is a gene that is mutated considerably in the smoking group in both the discovery and validation sets. Our previous research also repeatedly confirmed that TP53 mutations are associated with the better efficacy and prognosis of treating various tumors with ICIs (Lyu et al., 2020; Zhang Y. et al., 2020).

Based on a report on the close relationship between CNV and the occurrence and development of lung cancer (Qiu et al., 2017), we analyzed the difference in CNVs and found that the number of chromosome copy numbers changes (amplification or deletion) in the MSKCC data (Figure 3C) and TCGA data (Figure 3D), and was significantly higher in the smoking group. The significant detailed amplification or deletion sites among different groups are shown in Supplementary Figures 1A–D. This feature of more unstable chromosomes in the smoking group may be an essential factor in that smoking is more likely to cause tumors. Next, we conducted a separate analysis of the chromosome fragments that only changed in the smoking group (Figures 3E,F) and found that 19q13.2 (ACTN4), 19q12 (CCNE1), 8q24.3 (BAI1), 2q31.2 (ATP5G3), 17q12 (ACACA), and 14q32.33 (AKT1) chromosomal fragments had been significantly amplified in the smoking group in both data sets, where the 19q chromosome shows expansion of two arms. Together, 4q35.2 (SLC25A4), 2q37.3 (AGXT), 1p12 (ADORA3), 1q36.11 (RUNX3), 6q26 (ACAT2), 4p16.3 (ADD1), and 12q24.33 (ACAD5) chromosome fragments are significantly deleted in the smoking groups in both datasets, while chromosome 4 also showed changes in multiple arms.

These results indicate that the smoking group is more likely to comprise driver gene mutations and changes in chromosome copy number, which facilitates tumor formation in patients. Moreover, these changes are the likely reasons for the improved entry points and clinical prognosis of our immunotherapy.

### Comparison of Immune Characteristics Between Smoking and Non-smoking Groups

**FIGURE 4:**
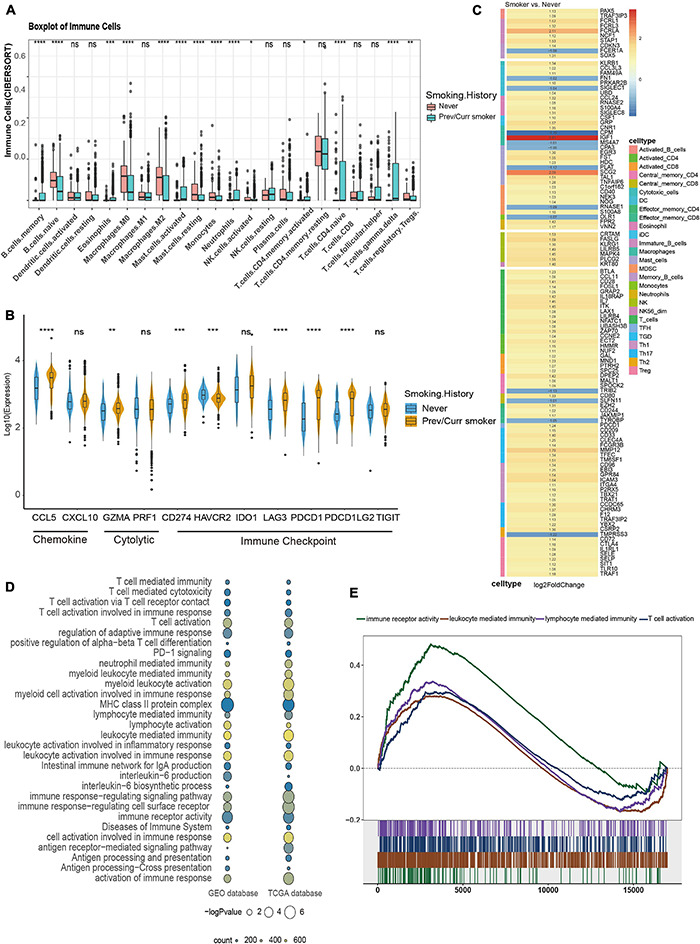
Comparison of immune characteristics between different smoking status in NSCLC. **(A)** CIBERSORT analyses quantifying the proportion of 22 immune cells in the different smoking status of the NSCLC cohort from TCGA. **(B)** Frequencies of stimulatory immunomodulators in the different smoking status of the NSCLC cohort from TCGA. **(C)** Heatmap showing average changes in the expression levels of immune-related gene between the previous or current smokers and non-smokers in the TCGA cohort. The genes corresponding to the same lymphocyte or function are identified by the same color on the left side of the squares, and each square with an exact number represents the logFC of a gene, filled with different back colors, i.e., from red to gray. The logFC values marked in black font indicate that the absolute value of logFC is ≥ 1 with statistical significance (*p* < 0.05). **(D)** The bubble chart shows that immune-related pathways are significantly different under different smoking states of TCGA and GEO dataset, the color of the circles indicates counts, as shown in the legend, and the size is proportional to the statistical significance. **(E)** GSEA of the hallmark gene sets downloaded from MSigDB. All transcripts were ranked by the log2 (fold change) between previous or current smokers and non-smokers in the TCGA cohort. Each run was performed with 1,000 permutations; immune-related pathways are highly enriched in the smoking group of TCGA cohort. *p < 0.05; **p < 0.01; ***p < 0.001; ****p < 0.0001; ns, not significant.

To further analyze the reasons for the difference in the efficacy of ICIs between the smoking group and the non-smoking group, we further analyzed the immune cell infiltration pattern between the two groups. Since the MSKCC immunotherapy cohort lacks RNA-seq data, we introduce the GEO’s GSE41271 data as the second validation set. Then, CIBERSORT software was used to analyze the infiltration abundance of 22 immune cells. The results of the TCGA data set showed that immune cells related to immune activation, such as activated CD4^+^ T cells, gamma delta T cells, and monocytes, were elevated significantly in the smoking group. In contrast, immune cells are related to immune suppression, such as M2 macrophages and regulatory T cells in the non-smoking group that appeared to aggregate (Figure 4A), and this result has also been verified using the GEO database (Supplementary Figure 2A). In addition, some stimulating immune modulators such as chemokines (CXCL5, CXCL10), cytolytic activity-related genes (PRF1, GZMA), and immune checkpoint biomarkers (CD274, IDOI) were significantly upregulated in the smoking group according to the analysis of related immune factors (Figure 4B). This result can also be seen in the same trend in GEO data (Supplementary Figure 2B).

Subsequently, we provided the immune cell–related marker gene table based on the study by Thorsson et al. (2018). We further analyzed the difference in immune infiltration patterns between smoking and non-smoking groups in the validation set. Through the differential analysis of immune-related genes in the TCGA database and GEO database, in the TCGA database 113 immune cell–related genes with significant differential expression were found, while in the GEO database 28 significant differences were found in genes. The TCGA heatmap showed that most of the 113 genes with significant differences were T cell–related markers, which were significantly higher in the smoking group (Figure 4C). The heatmap of the 28 substantially different genes from the GEO database also shows that most of the immune-related genes with significant differences are activated CD4^+^ T cells and activated CD8^+^ T cell markers, which are highly expressed in the smoking group (Supplementary Figure 2C). We also found that seven genes were simultaneously verified in both databases, and six of them were highly expressed in the smoking group. Among them, HMMR, GAL, and SPC25 are signs of activated T cells (Supplementary Figures 2D,E).

At the same time, we used the “clusterProfiler” package to perform a GSEA on the TCGA and the GEO datasets. The results found that pathways related to the positive regulation of immune response such as immune response to tumor cells, T cell activation–related pathways, inflammatory reactions, natural killer cell–mediated cytotoxicity, MHC class Ib for antigen processing and presentation, and positive regulation of MHC class II biosynthesis processes were significantly enriched in the smoking group (Figures 4D,E). In short, our analysis of the immune infiltration patterns between different NSCLC groups revealed that the smoking group has a higher abundance of immune cells and immune factor infiltration than the non-smoking group. These data further explain why patients in our smoking group experienced improved efficacy when treated with ICIs than in the non-smokers with NSCLC.

### The Mass Cytometry Analysis Indicated That the Smoking Group Had an Activated Immune Microenvironment

The immune microenvironment in NSCLC has been widely studied (Conforti et al., 2021); however, the immune microenvironment of different smoking status in NSCLC has not been systematically analyzed before. We collected fresh tumor tissues from eight NSCLC patients from Zhujiang Hospital of Southern Medical University for CyTOF analysis. After quality control, we obtained 1,277,343 cells (an average of ≈250,000 cells per sample). Based on the expression of CD3, CD19, CD68, CD14, and other markers, they were annotated as T cells, B cells, and myeloid cells, respectively. After preliminary annotations, each type of immune cell was re-clustered, grouped, and combined with the expression of 42 surface markers. The cells were divided into different subtypes. The flow chart of this process is shown in Figure 1, and the sample quality control chart, the number of cells in each sample, and the basic expression heat map of the 42 markers in each sample are shown in Supplementary Figure 3.

#### All Immune Cell Groups

**FIGURE 5:**
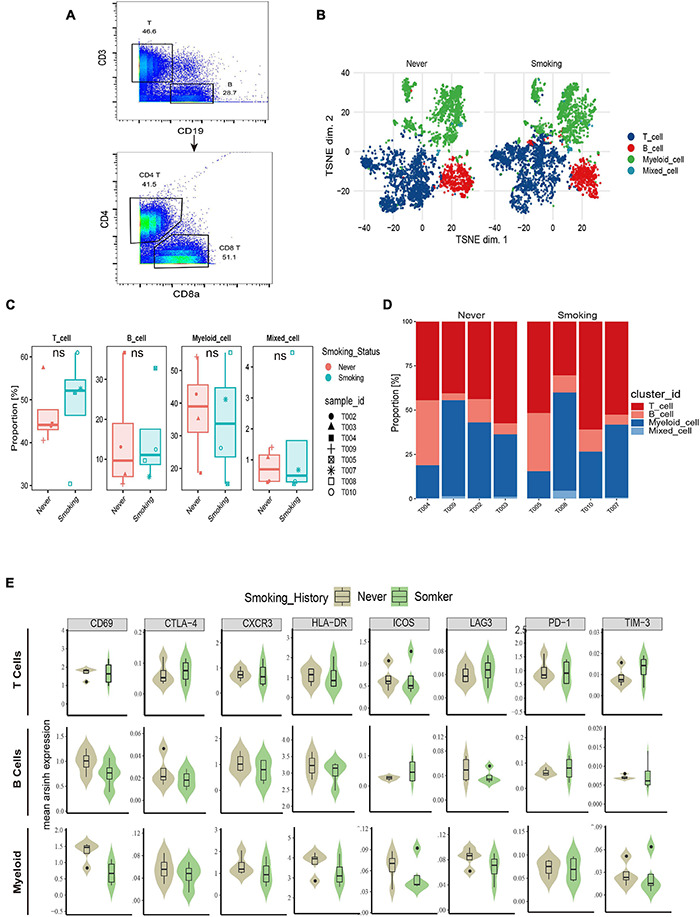
The immune landscape of NSCLC patients sequenced by CyTOF. **(A)** Schematic diagram of flow gating. CD45^+^ cells were manually selected and subjected to sequential gating to identify the Immune cell subsets with CyTOF. **(B)** t-SNE plot showing the overall distribution difference of immune clusters between previous or current smokers and non-smokers. **(C)** Box plot comparing the relative abundance of each immune cluster between different smoking status groups. Box plot center and box correspond to median and IQR, respectively. Different shapes were used to represent each patient. **(D)** Bar plot showing the relative abundance of 4 immune cell types in each sample, faceted by smoking status. **(E)** The expression of surface molecules showed in different immune lineages.

According to the immune cell markers, CD45^+^ cells were divided using the manual gated circle function of the Flowjo (Figure 5A), and multi-dimensional data are converted into single-cell two-dimensional visualization data by R package cytofworkflow.

The overall immune lineage is divided into T cells (CD3^+^), B cells (CD19^+^), and myeloid cells (CD3^–^CD19^–^CD56^–^CD11b^+^), visualized in the tSNE diagram (Figure 5B). In the tumor immune microenvironment of all NSCLC samples, the proportion of T cells is the highest, followed by myeloid cells and B cells (Supplementary Figure 4A). Notably, T cells related to immune regulation and tumor killing in the smoking group were higher than that in the non-smoking group, and the proportion of myeloid cells was larger in the non-smoking group; there was no significant difference in B cells between the two groups (Figures 5C,D). Diversified expression patterns of surface markers were observed in different immune cell lineages from different smoking status (Figure 5E). These results preliminarily indicate the difference in immune lineage distribution between smoking and non-smoking groups.

#### CD4^+^ T Cell Clustering

**FIGURE 6:**
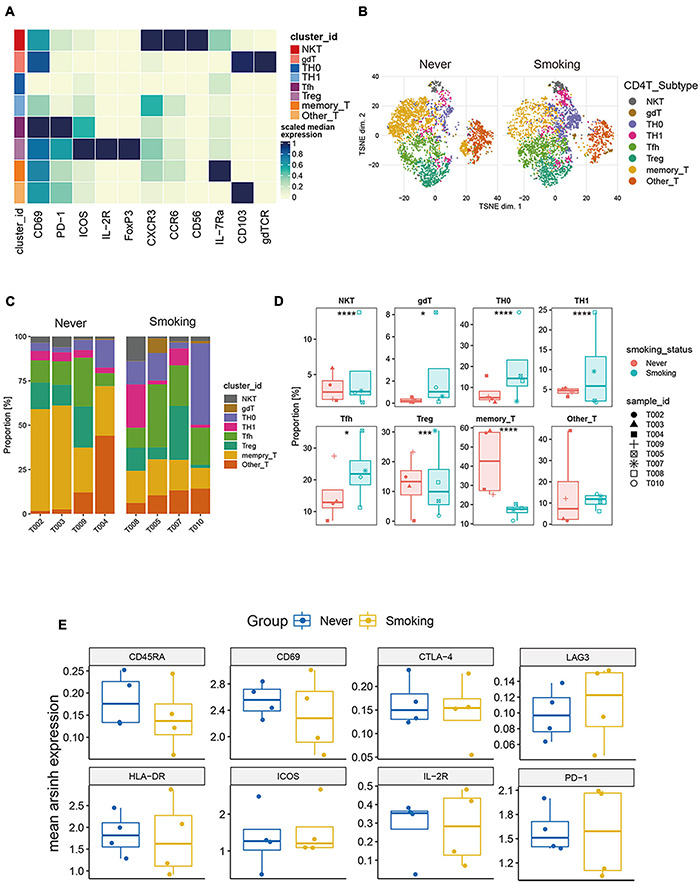
Features of CD4^+^ T cells in NSCLC with different smoking status. **(A)** Heatmap of the median marker intensities of the multiple lineage markers in the 8 CD4^+^ T cell populations obtained by manual merging of the 30 metaclusters generated by FlowSOM. The heat represents the median of arcsinh and 0–1 transformed marker expression calculated over cells from all the samples. **(B)** t-SNE plot showing the overall distribution of different CD4^+^ T cell clusters between previous/current smokers and non-smokers. **(C)** Bar plot showing the relative abundance of different CD4^+^ T cell types in each sample, faceted by smoking status. **(D)** Box plot comparing the relative abundance of each CD4^+^ T cell cluster between different smoking status groups. Box plot center and box correspond to median and IQR, respectively. Different shapes were used to represent each patient. *p < 0.05; **p < 0.01; ***p < 0.001; ****p < 0.0001; ns, not significant. **(E)** The expression level of several functional markers in different groups.

To explore the heterogeneity of the composition of CD4^+^ T cell subgroups between different groups, we carried out re-clustering and downstream analysis of the CD4^+^ T cell subgroups (Figure 6A). CD4^+^ T cells are further divided into eight immune cell subgroups, including follicular helper CD4^+^ T cells, gamma delta CD4^+^ T cells, CD4^+^ Tregs, NKT cells, Th0 CD4^+^ T cells, Th1 CD4^+^ T cells, memory CD4^+^ T cells, and other CD4^+^ T cells, visualized in the tSNE diagram (Figure 6B). We also visualized the expression of markers used to annotate CD4^+^ T cell subsets in different groups (Supplementary Figures 5A,B).

Among them, the proportion of memory CD4^+^ T cells was the highest, and the proportion of NKT cells in all CD4^+^ T cells was relatively small (Supplementary Figure 4B). Then, we compared the proportions of different types of CD4^+^ T cells between the smoking and non-smoking groups (Figures 6C,D), and found that T cell infiltration that promotes the positive immune response is mainly used in the smoking group. This phenomenon included follicular helper T cells (Tfh), NKT, Th0, Th1, and gamma delta T cells (gdT). The proportions of these cells are significantly higher in the smoking group (*p* < 0.05, Figure 6D), while the proportion of Treg (regulatory T cells) with immunosuppressive effects and memory CD4^+^ T cells was significantly higher in the non-smoker group (*p* < 0.05, Figure 6D).

Then, we compared the expression levels of immune exhaustion and activation markers between different groups (Figure 6E). The expression of CD45RA can be seen slightly higher in the non-smoking group than the smoking group, which indicates that the proportion of CD45RA^high^ CD4^+^ T cells in the non-smoking group may be greater. However, the expression patterns of exhaustion markers including TIM-3, PD-1, CTLA-4, and LAG-3, and activation markers including ICOS, CD69, and HLA-DR in different groups of CD4^+^ T cells had no difference. This result was a bit different from the conclusion that activated CD4^+^ T cells are more abundant in the smoking group in the analysis of the transcriptome in TCGA and GEO sets. We consider that it is due to the small sample size of CyTOF data and individual differences.

#### CD8^+^ T Cell Clustering

**FIGURE 7:**
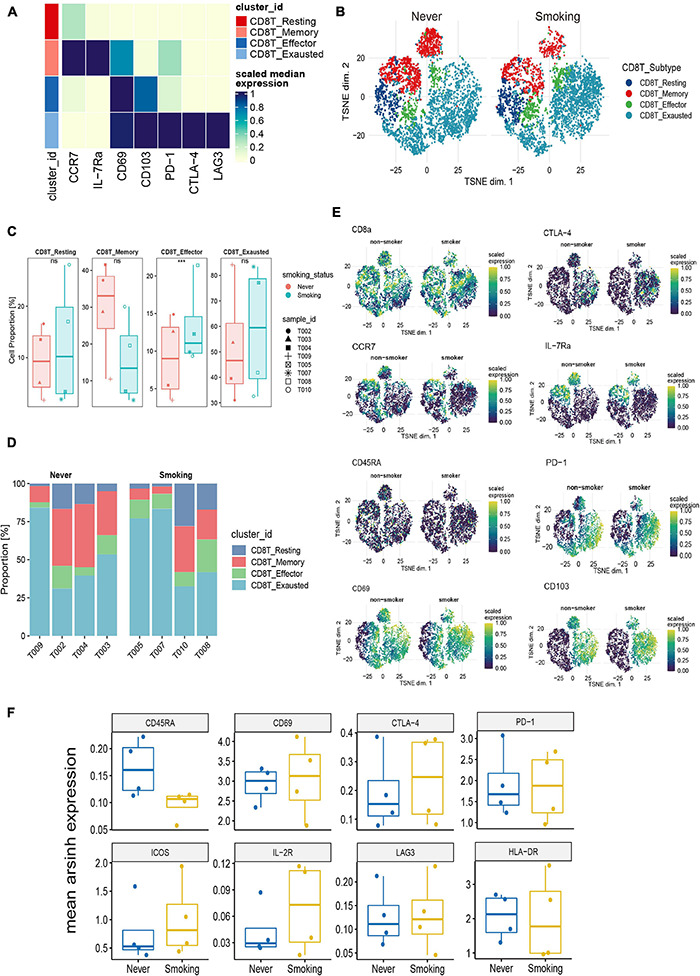
Features of CD8^+^ T cells in NSCLC with different smoking status. **(A)** Heatmap of the median marker intensities of the multiple lineage markers in the 4 CD8^+^ T cell populations obtained by manual merging of the 30 metaclusters generated by FlowSOM. The heat represents the median of arcsinh and 0–1 transformed marker expression calculated over cells from all the samples. **(B)** t-SNE plot showing the overall distribution of different CD8^+^ T cell clusters between previous/current smokers and non-smokers. **(C)** Box plot comparing the relative abundance of each CD8^+^ T cell cluster between different smoking status groups. Box plot center and box correspond to median and IQR, respectively. Different shapes were used to represent each patient. *p < 0.05; **p < 0.01; ***p < 0.001; ****p < 0.0001; ns, not significant. **(D)** Bar plot showing the relative abundance of different CD8^+^ T cell types in each sample, faceted by smoking status. **(E)** t-SNE plots of markers used to annotate CD8^+^ T subgroups in different groups. **(F)** The expression level of several functional markers in different groups.

To illustrate the differences in CD8^+^ T cells in different smoking states, we further re-clustered CD8^+^ T cells. According to existing markers, CD8^+^ T cells are further divided into four immune cell subgroups (Figure 7A). The tSNE chart shows that the exhausted CD8^+^ T cells make up the largest proportion of NSCLC tissue, and the proportion of effector CD8^+^ T cells was relatively small (Supplementary Figure 4C). Then, we compared the proportions of different types of CD8^+^ T cells between the smoking and non-smoking groups and found that, compared with those in the non-smoking group, the level of effector CD8^+^ T cells was significantly upregulated (*p* < 0.001; Figures 7B,C) in the smoking group. Although exhausted CD8^+^ T cells accounted for the largest proportion, there was no significant difference between different groups (Figure 7C). In addition, Figure 7D was used to show the proportion of each cell type in each sample in detail. We also discovered that expression of surface markers, including CD8a, CTLA-4, CCR7, CD69, CD45RA, IL-7Ra, PD-1, and CD103, were different in smokers and non-smokers (Figure 7E). The representative expression patterns of function surface markers found that CD8^+^ T cells in the non-smoking group exhibited a rest state with increased CD45RA expression and low expression of HLA-DR and CD8^+^ T cells in the smoking group showed an activation state with an increased expression of activation markers (HLA-DR, ICOS, IL-2R) (Figure 7F).

#### Clustering of Myeloid Cells

**FIGURE 8:**
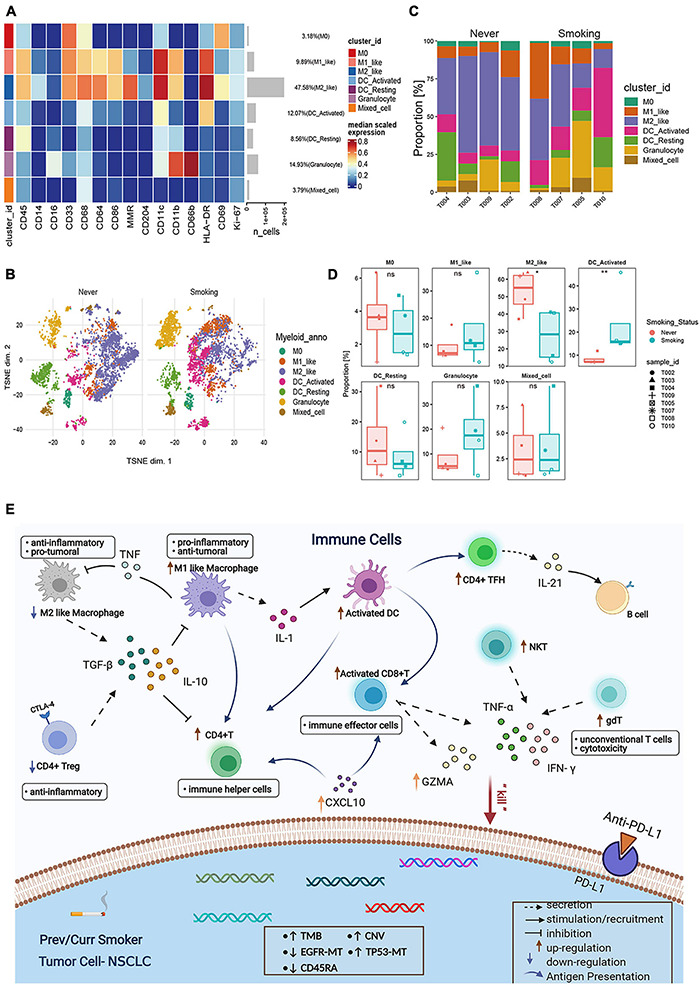
Features of myeloid cells in NSCLC with different smoking status. **(A)** Heatmap of the median marker intensities of the multiple lineage markers in the 7 myeloid cell populations obtained by manual merging of the 30 metaclusters generated by FlowSOM. The heat represents the median of arcsinh and 0–1 transformed marker expression calculated over cells from all the samples. **(B)** t-SNE plot showing the overall distribution of different myeloid cell clusters between previous/current smokers and non-smokers. **(C)** Bar plot showing the relative abundance of different myeloid cell types in each sample, faceted by smoking status. **(D)** Box plot comparing the relative abundance of each myeloid cell cluster between different smoking status groups. Box plot center and box correspond to median and IQR, respectively. Different shapes were used to represent each patient. *p < 0.05; **p < 0.01; ***p < 0.001; ****p < 0.0001; ns, not significant. **(E)** Possible mechanism for the improved prognosis after immunotherapy of smoking patients in NSCLC. Overview of the key immune cell population changes in previous or current smokers compared with non-smokers. Different immune cell types and their potential connections through cytokines, chemokines, or receptor–ligand interactions are shown in this picture. In addition, the genomic changes were also plotted inside the tumor cell. TMB, tumor mutational burden; CNV, copy number variant; MT, mutation type. Figure created with BioRender.com.

In addition to T cells, myeloid cells are also important immune cells that exert anti-tumor effects and participate in many aspects of anti-tumor immunity (Cheng et al., 2021). Therefore, to explore the differences between myeloid cell populations in the NSCLC of previous or current smokers, and non-smokers, we re-clustered and re-analyzed the myeloid cells (Figure 8A). Macrophages accounted for the largest proportion of myeloid cells in the tumor microenvironment, and immunosuppressive M2 macrophages accounted for the largest proportion in macrophages (Supplementary Figure 4D). Furthermore, a comparative analysis of the proportions of different types of myeloid cells between the smoking and non-smoking groups (Figures 8B,C) found that the proportion of activated DC that exerts a positive immunomodulatory effect in the former was significantly higher in the group of smokers than in non-smokers (*p* < 0.01, Figure 8D), and M2 type macrophages in the non-smoking group were significantly higher (*p* < 0.05, Figure 8D). This result is consistent with the previous results of the TCGA and GEO’s CIBERSORT.

In summary, the CyTOF results further verify that the smoking group has an activated immune microenvironment from the proportion of immune cell infiltration. In comparison, the immune microenvironment of the non-smoking group is in a suppressed or rest state. These results can also be verified from the TCGA and GEO transcriptome data.

## Discussion

The efficacy ICIs of NSCLC in different populations is variable, and smoking status has also become one indicator for judging the efficacy of ICIs (Gainor et al., 2020). However, the underlying mechanism of the difference in the efficacy of treatment with ICIs between smokers and non-smokers remains unclear. Our research further elaborated on the relationship between smoking and treatment with ICIs from multiple MSKCC, TCGA, GEO, and CyTOF data levels. It explained the underlying reasons for its differences from various aspects such as mutations, CNVs, the transcriptome, and the immune microenvironment.

We performed a Cox regression and survival analysis from the clinical data, and found that the smoking group was associated with longer PFS in patients treated with ICIs. Next, we analyzed the possible mechanism leading to differences in the efficacy of ICIs between smoking and non-smoking patients. We found high TMB levels and neoantigen loads in the smoking group. Subsequently, from MSKCC and TCGA, we found a higher frequency of gene mutations (TP5, KRAS, MUC16) in the smoking group compared with the non-smoking group and a higher level of CNVs. Driver gene mutations, especially TP53 mutations and higher CNVs, are related to the better efficacy of ICIs. These high-frequency mutations and increased CNVs can activate pathways like cell metabolism, and regulate angiogenesis, T cells, and antigen expression. In turn, these may activate the immune microenvironment through direct or indirect relationships, thereby affecting the efficacy of ICIs (Yi et al., 2020; Zhang J. et al., 2020; Si et al., 2021).

Subsequently, we introduced a second validation set from the GEO database GSE41271 as an auxiliary verification of the TCGA validation set to compare the immune microenvironment between the different groups. We learned from the immune infiltrating cells and immune-related genes (antigen presentation/stimulation/inhibition), immune cell–related genes, and immune-related pathways in our comprehensive analysis. This study is also the first to evaluate the immune microenvironment of smoking and non-smoking from NSCLC as a whole, and found that smoking group’s immune microenvironment was activated in both validation sets, including high expression of immune infiltrating M1 macrophages, monocytes, and activated CD4^+^ T cells; high immune positive regulatory chemokine expression, cytolytic activity–related genes, and immune checkpoint biomarkers; high expression of activated CD4^+^/CD8^+^ T cell–related genes HMMR and GAL; and the activation of immune-related pathways (T cell activation–related pathways, inflammatory response, natural killer cell–mediated cytotoxicity). A few studies have shown that the stimulating effect of tobacco smoke on the respiratory tract can lead to the release of pro-inflammatory cytokines, such as TNF-α, IL-1, IL-6, IL-8, and granulocyte-macrophage colony-stimulating factors. In turn, the number of leukocytes can be increased (such as T cells, natural killer cells, and monocytes), and the unstable T cells in constant circulation cause continuous and permanent inflammatory damage to normal lung tissue because of long-term exposure to smoke from cigarettes (Chen et al., 2016; Piaggeschi et al., 2021). In our NSCLC patients who were also smokers, the increased immune cells and activated immune microenvironment may instead become the target of ICI therapy.

Next, we collected eight fresh NSCLC cancer tissues for CyTOF to further analyze the difference in the immune infiltration pattern between the smoking and non-smoking groups. Since T cells play one of the most critical roles in the tumor microenvironment through their anti-tumor effect, they are also important target cells for tumor therapy based on the immune microenvironment (Binnewies et al., 2018). Our analysis focused on comparing different types of CD4^+^ T cells and CD8^+^ T cell infiltration, and found that 80% of T cells related to positive immunomodulation (such as Tfh, gdT, NKT, Th1, and activated CD8^+^ T cells) are highly expressed in previous or current smokers, while Treg cells related to immunosuppression were highly enriched in the non-smoking group.

At the same time, we found that CD45RA was highly expressed in CD4^+^ T and CD8^+^ T cells in the non-smoker group. As we all know, naïve T cells express CD45RA and are usually functionally quiescent (Sallusto et al., 2004). Huang et al. (2015) also found a similar result in APC that lower levels of CD4^+^ naïve/memory ratio were positively correlated with better OS (*p* = 0.036 and 0.021, respectively), and CD8^+^ naïve/memory ratio can be a candidate marker for predicting PFS and its change may reflect the progression. Therefore, the CD45RA^high^CD4^+^ T cells and CD45RA^high^CD8^+^ T cells enriched in the non-smoking group may also be one of the reasons for the poor prognosis of ICIs in the non-smoking group.

For the analysis of myeloid cells, we found that M2 macrophages were highly enriched in the non-smoker group. M2 macrophages in myeloid cells have always been the cell type that researchers have focused on. They secrete various immunosuppressive chemokines, cytokines, and extracellular matrix components; they also negatively regulate immune response while reshaping immune microenvironment and promoting tumor progression and metastasis (Han et al., 2021). In our study, the high infiltration of M2-type macrophages in non-smokers may also be a fundamental reason for their poor prognosis. Also, studies have shown M1 macrophages’ high degree of infiltration is associated with better immune efficacy metastatic urothelial carcinoma (Zeng et al., 2020). This outcome also suggests that the high M1 macrophage infiltration in smoking patients in NSCLC may be transformed into the benefit of immunotherapy.

We summarized the mechanism by which smoking NSCLC may affect the efficacy of ICIs (Figure 8E). In short, these results partly explain NSCLC in the smoking group’s better prognosis in the treatment of ICIs. It is likely the result of multiple factors working together and complement each other.

This study has some limitations. First, on the multivariate Cox regression analysis of clinical data from the MSKCC receiving ICIs cohort, we found that smoking was associated with the prognosis of ICI treatment, but it was not significant (HR = 0.67, 95% CI: 0.44–1.0, *p* = 0.059). Due to the lack of NSCLC cohorts receiving ICI treatment, we are unable to conduct further verification. Second, there is a lack of mRNA data for the patients in the NSCLC cohort treated with ICIs. In this set of findings, we could not directly assess whether the prognosis of ICIs is different due to differences in their immune characteristics and immune microenvironment. Third, due to the small number of collected samples, possible individual differences may make the results slightly different from the transcriptome results. Finally, due to the limitations of the selected markers, we had not been able to make more detailed annotations and studies on B cells. We still need to expand the sample size for deeper and more representative research.

## Conclusion

Our study confirmed that in NSCLC patients treated with ICIs, previous or current smokers have a better prognosis after treatment with ICIs than non-smokers. This outcome is the same as the smoking group, which had higher gene mutations, more copy number variations, and a stronger immune microenvironment. While smoking is one of the main risk factors for NSCLC, it is also an important indicator for predicting the efficacy of treatment with ICIs. NSCLC patients who are treated with ICIs in clinical practice may also consider smoking status as a key indicator for maximizing the benefits of treating patients with ICIs.

## Data Availability Statement

The datasets presented in this study can be found in online repositories. The names of the repository/repositories and accession number(s) can be found in the article/Supplementary Material. The CyTOF datasets generated during and/or analysed during the current study are available from the corresponding author on reasonable request.

## Ethics Statement

The studies involving human participants were reviewed and approved by the Zhujiang Hospital of Southern Medical University (Guangzhou, China). The patients/participants provided their written informed consent to participate in this study.

## Author Contributions

PL and JZ: conceptualization and supervision. YS: formal analysis. YS, QY, JS, TW, and WS: software. PL, JZ, and YS: resources. YS and QY: visualization. YS, QY, JS, TW, NZ, and WS: writing – original draft and writing, review, and editing. All authors contributed to the article and approved the submitted version.

## Conflict of Interest

The authors declare that the research was conducted in the absence of any commercial or financial relationships that could be construed as a potential conflict of interest.

## Publisher’s Note

All claims expressed in this article are solely those of the authors and do not necessarily represent those of their affiliated organizations, or those of the publisher, the editors and the reviewers. Any product that may be evaluated in this article, or claim that may be made by its manufacturer, is not guaranteed or endorsed by the publisher.

## Footnotes

1
https://www.cbioportal.org/study/summary?id=nsclc_pd1_msk_2018

2
https://www.cbioportal.org/study/summary?id=nsclc_tcga_broad_2016

3
https://portal.gdc.cancer.gov/

4
https://cloud.genepattern.org/gp/pages/index.jsf

5
http://cibersort.stanford.edu/

## Abbreviations

ICI: immune checkpoint inhibitor
NSCLC: non-small cell lung cancer
MSKCC: Memorial Sloan Kettering Cancer Center
TCGA: The Cancer Genome Atlas
GEO: Gene Expression Omnibus
CNV: copy number variation
CyTOF: mass cytometry
GDSC: Genomics of Drug Sensitivity in Cancer
CTRP: Cancer Therapeutics Response Portal
PRISM: Profiling Relative Inhibition Simultaneously in Mixtures
IC50: half maximal inhibitory concentration
AUC: area under the dose–response curve
PD-1: programmed cell death 1
PD-L1: programmed cell death ligand 1
CTLA4: cytotoxic T lymphocyte antigen 4
TMB: tumor mutation burden
MSI: microsatellite instability
dMMR: DNA mismatch repair
PFS: progression-free survival
DOR: duration of response
GEP: gene expression profile
OS: overall survival
GSEA: Gene Set Enrichment Analysis
GO: Gene Ontology
KEGG: Kyoto Encyclopedia of Genes and Genomes
TKI: tyrosine-kinase inhibitors
Tfh: follicular helper T cells
gdT: gamma delta T cells
Treg: regulatory T cells
IFN gamma: interferon-gamma
TNF: tumor necrosis factor.
