# Supplementary material for: The Effect of Smoking on the Immune Microenvironment and Immunogenicity and Its Relationship With the Prognosis of Immune Checkpoint Inhibitors in Non-small Cell Lung Cancer
Source: Front Cell Dev Biol. 2021 Sep 28;9:745859. doi: 10.3389/fcell.2021.745859 (PMC8512705; doi:10.3389/fcell.2021.745859)
Supplement: Supplementary file 1 [file Data_Sheet_1.docx]

**Supplement Figure Legends:**

Table S1.Chemical reagents used in CyTOF

| **List** | **Isotopes** | **Antibodies(Human)** | **Clone** | **Catalogue Number** | **Source** |
| --- | --- | --- | --- | --- | --- |
| 1 | **89Y** | CD45 | HI30 | 304002 | BioLegend |
| 2 | **115In** | CD3 | UCHT1 | BE0231 | Bio Cell |
| 3 | **139La** | CD68 | Y1/82A | 333802 | BioLegend |
| 4 | **141Pr** | CD56 | NCAM16.2 | 559043 | BD |
| 5 | **142Nd** | gdTCR | 5A6.E9 | PLTTECH | PLTTECH |
| 6 | **143Nd** | CD196/CCR6 | G034E3 | 353402 | BioLegend |
| 7 | **144Nd** | CD14 | M5E2 | 301810 301862 | BioLegend |
| 8 | **145Nd** | CD103 | B-Ly7 | 14-1038-82 | eB |
| 9 | **146Nd** | CD123/IL-3R | 6H6 | 306002 | BioLegend |
| 10 | **147Sm** | CD366/TIM-3 | F38-2E2 | 345004 345010 | BioLegend |
| 11 | **148Nd** | CD19 | HIB19 | 302214 | BioLegend |
| 12 | **149Sm** | CD25/IL-2R | 24212 | MAB1020 | RD |
| 13 | **150Nd** | CD274/PD-L1 | 29E.2A3 | 329702 329716 | BioLegend |
| 14 | **151Eu** | CD278/ICOS | C398.4A | 313502 | BioLegend |
| 15 | **152Sm** | CD39 | A1 | 328202 | BioLegend |
| 16 | **153Eu** | CD204 | 351615 | MAB2708 | RD |
| 17 | **154Sm** | CD169 | 7-239 | 346002 | BioLegend |
| 18 | **155Gd** | CD45RA | HI100 | 304102 | BioLegend |
| 19 | **156Gd** | CD86 | Fun-1 | 555655 | BD |
| 20 | **157Gd** | CD28 | CD28.2 | 302934 | BioLegend |
| 21 | **158Gd** | CD197/CCR7 | G043H7 | 353222 353256 | BioLegend |
| 22 | **159Tb** | CD11c | BU15 | 337202 | BioLegend |
| 23 | **160Gd** | CD33 | WM53 | 303419 | BioLegend |
| 24 | **161Dy** | CD152(CTLA-4) | 14D3 | 14-1529-82 | eB |
| 25 | **162Dy** | FoxP3 | PCH101 | 14-4776-82 | eB |
| 26 | **163Dy** | CD163 | GHI/61 | 333602 | BioLegend |
| 27 | **164Dy** | CD64 | 10.1 | 305016 305002 | BioLegend |
| 28 | **165Ho** | CD66b | G10F5 | 305102 | BioLegend |
| 29 | **166Er** | CD183/CXCR3 | G025H7 | 353750 | BioLegend |
| 30 | **167Er** | CD206/MMR | 15-2 | 321112 321150 | BioLegend |
| 31 | **168Er** | CD69 | FN50 | 310902 | BioLegend |
| 32 | **169Tm** | Ki-67 | SolA15 | 14-5698-82 | eB |
| 33 | **170Er** | CD127/IL-7Ra | A019D5 | 351302 | BioLegend |
| 34 | **171Yb** | CD279/PD-1 | EH12.2H7 | 329926 | BioLegend |
| 35 | **172Yb** | CD38 | HIT2 | 303502 | BioLegend |
| 36 | **173Yb** | Granzyme B | QA16A02 | 372202 | BioLegend |
| 37 | **174Yb** | CD223/LAG3 | 874501 | MAB23193 | RD |
| 38 | **175Lu** | CD16 | 3G8 | 302014 | BioLegend |
| 39 | **176Yb** | HLA-DR | L243 | 307612 307648 | BioLegend |
| 40 | **197Au** | CD4 | RPA-T4 | 300516 | BioLegend |
| 41 | **198Pt** | CD8a | RPA-T8 | 301018 301074 | BioLegend |
| 42 | **209Bi** | CD11b | M1/70 | 101202 | BioLegend |

Table S2.Patients characteristics of the MSKCC database

|  | **Never (N=47)** | **Prev/Curr**  **(N=193)** | **Overall (N=240)** |
| --- | --- | --- | --- |
| **Age** |  |  |  |
| Mean (SD) | 60.4 (15.4) | 65.4 (10.1) | 64.4 (11.5) |
| Median [Min, Max] | 62.0[22.0, 92.0] | 66.0 [37.0, 88.0] | 66.0 [22.0, 92.0] |
| **Sex** |  |  |  |
| Female | 29 (61.7%) | 93 (48.2%) | 122 (50.8%) |
| Male | 18 (38.3%) | 100 (51.8%) | 118 (49.2%) |
| **Type** |  |  |  |
| Large Cell Neuroendocrine Carcinoma | 0 (0%) | 7 (3.6%) | 7 (2.9%) |
| Lung Adenocarcinoma | 41 (87.2%) | 145 (75.1%) | 186 (77.5%) |
| Lung Squamous Cell Carcinoma | 6 (12.8%) | 28 (14.5%) | 34 (14.2%) |
| Non-Small Cell Lung Cancer | 0 (0%) | 13 (6.7%) | 13 (5.4%) |
| **Tumor mutation burden** |  |  |  |
| Mean (SD) | 4.49 (2.82) | 11.9 (11.1) | 10.4 (10.5) |
| Median [Min, Max] | 4.00 [1.00, 14.0] | 9.00 [1.00, 89.0] | 7.50 [1.00, 89.0] |
| **PD-L1 Score(%)** |  |  |  |
| Mean (SD) | 9.41 (24.8) | 25.7 (35.7) | 22.5 (34.3) |
| Median [Min, Max] | 0 [0, 75.0] | 3.00 [0, 100] | 1.00 [0, 100] |
| Missing | 30 (63.8%) | 124 (64.2%) | 154 (64.2%) |
| **ICI Treatment** |  |  |  |
| Combination | 9 (19.1%) | 25 (13.0%) | 34 (14.2%) |
| Monotherapy | 38 (80.9%) | 168 (87.0%) | 206 (85.8%) |
| **Overall.Survival(month)** |  |  |  |
| Mean (SD) | 19.3 (16.5) | 16.0 (12.0) | 16.6 (13.0) |
| Median [Min, Max] | 14.5 [0, 57.0] | 14.0 [1.00, 57.0] | 14.0 [0, 57.0] |
| Missing | 11 (23.4%) | 36 (18.7%) | 47 (19.6%) |
| **Progression Free Survival(month)** |  |  |  |
| Mean (SD) | 4.32 (4.75) | 5.90 (6.15) | 5.59 (5.93) |
| Median [Min, Max] | 2.60 [0.370, 24.3] | 3.60 [0.600, 30.5] | 3.17 [0.370, 30.5] |

Abbreviations:ICI: immune checkpoint inhibitor; Never:never smoker; Prev/Curr: previous or current smoker

Table S3.Patients characteristics of the TCGA database

|  | **Never  (N=111)** | **Prev/Curr  (N=976)** | **Overall (N=1087)** |
| --- | --- | --- | --- |
| **Age** |  |  |  |
| Mean (SD) | 65.7 (9.84) | 66.5 (9.24) | 66.4 (9.30) |
| Median [Min, Max] | 65.0 [39.0, 84.0] | 68.0 [38.0, 90.0] | 67.0 [38.0, 90.0] |
| Missing | 26 (23.4%) | 129 (13.2%) | 155 (14.3%) |
| **Sex** |  |  |  |
| Female | 77 (69.4%) | 370 (37.9%) | 447 (41.1%) |
| Male | 34 (30.6%) | 606 (62.1%) | 640 (58.9%) |
| **Cancer.Type** |  |  |  |
| Lung Adenocarcinoma | 93 (83.8%) | 521 (53.4%) | 614 (56.5%) |
| Lung Squamous Cell Carcinoma | 18 (16.2%) | 455 (46.6%) | 473 (43.5%) |
| **Tumor mutation burden** |  |  |  |
| Mean (SD) | 2.25 (3.05) | 6.26 (5.68) | 5.85 (5.60) |
| Median [Min, Max] | 1.16 [0.0222, 20.1] | 4.77 [0.0444, 59.4] | 4.49 [0.0222, 59.4] |
| **Stage** |  |  |  |
| I | 2 (1.8%) | 6 (0.6%) | 8 (0.7%) |
| IA | 25 (22.5%) | 215 (22.0%) | 240 (22.1%) |
| IB | 30 (27.0%) | 288 (29.5%) | 318 (29.3%) |
| II | 1 (0.9%) | 2 (0.2%) | 3 (0.3%) |
| IIA | 11 (9.9%) | 112 (11.5%) | 123 (11.3%) |
| IIB | 18 (16.2%) | 149 (15.3%) | 167 (15.4%) |
| III | 0 (0%) | 3 (0.3%) | 3 (0.3%) |
| IIIA | 15 (13.5%) | 134 (13.7%) | 149 (13.7%) |
| IIIB | 2 (1.8%) | 29 (3.0%) | 31 (2.9%) |
| IV | 6 (5.4%) | 30 (3.1%) | 36 (3.3%) |
| Missing | 1 (0.9%) | 8 (0.8%) | 9 (0.8%) |
| **Overall.Survival(month)** |  |  |  |
| Mean (SD) | 9.92 (14.9) | 19.8 (27.6) | 18.9 (26.8) |
| Median [Min, Max] | 3.90 [0, 70.8] | 8.55 [0, 224] | 7.80 [0, 224] |
| Missing | 26 (23.4%) | 130 (13.3%) | 156 (14.4%) |
| **Progression Free Interval(month)** |  |  |  |
| Mean (SD) | 19.9 (18.2) | 27.0 (29.1) | 26.4 (28.3) |
| Median [Min, Max] | 15.5 [0.133, 109] | 18.4 [0.0333, 242] | 18.1 [0.0333, 242] |
| Missing | 27 (24.3%) | 137 (14.0%) | 164 (15.1%) |

Abbreviations:Never:never smoker; Prev/Curr: previous or current smoker

Table S4.Patients characteristics of the GSE41271

|  | **Never  (N=28)** | **Prev/Curr  (N=243)** | **Overall (N=271)** |
| --- | --- | --- | --- |
| **Sex** |  |  |  |
| Female | 19 (67.9%) | 107 (44.0%) | 126 (46.5%) |
| Male | 9 (32.1%) | 136 (56.0%) | 145 (53.5%) |
| **Stage** |  |  |  |
| IA | 7 (25.0%) | 41 (16.9%) | 48 (17.7%) |
| IA Vs IIIB | 0 (0%) | 1 (0.4%) | 1 (0.4%) |
| IB | 10 (35.7%) | 74 (30.5%) | 84 (31.0%) |
| IIA | 0 (0%) | 11 (4.5%) | 11 (4.1%) |
| IIB | 3 (10.7%) | 36 (14.8%) | 39 (14.4%) |
| IIIA | 3 (10.7%) | 45 (18.5%) | 48 (17.7%) |
| IIIB | 2 (7.1%) | 32 (13.2%) | 34 (12.5%) |
| IV | 3 (10.7%) | 3 (1.2%) | 6 (2.2%) |
| **Type** |  |  |  |
| Adenocarcinoma | 26 (92.9%) | 156 (64.2%) | 182 (67.2%) |
| Adenosquamous | 0 (0%) | 2 (0.8%) | 2 (0.7%) |
| LCC-NE | 0 (0%) | 3 (1.2%) | 3 (1.1%) |
| NSCLC | 0 (0%) | 1 (0.4%) | 1 (0.4%) |
| Pleomorphic carcinoma | 0 (0%) | 1 (0.4%) | 1 (0.4%) |
| Sarcomatoid | 1 (3.6%) | 1 (0.4%) | 2 (0.7%) |
| Sarcomatoid-Adenocarcinoma | 0 (0%) | 1 (0.4%) | 1 (0.4%) |
| Sarcomatoid-squamous | 0 (0%) | 1 (0.4%) | 1 (0.4%) |
| Squamous | 1 (3.6%) | 77 (31.7%) | 78 (28.8%) |
| **Race** |  |  |  |
| African American | 1 (3.6%) | 14 (5.8%) | 15 (5.5%) |
| Asian | 6 (21.4%) | 1 (0.4%) | 7 (2.6%) |
| Caucasian | 17 (60.7%) | 224 (92.2%) | 241 (88.9%) |
| Hispanic | 4 (14.3%) | 4 (1.6%) | 8 (3.0%) |

Abbreviations:Never:never smoker; Prev/Curr: previous or current smoker

Table S5.Patients characteristics of the CyTOF database

| **ID** | Sex | Age（year） | Histology | Stage | Smoking | EGFR | ALK | PD-L1 | BMI |
| --- | --- | --- | --- | --- | --- | --- | --- | --- | --- |
| **T002** | female | 67 | Adenocarcinoma | IA | Never | / | (-) | 30% | 19.56 |
| **T003** | female | 37 | Adenocarcinoma | IIB | Never | / | (-) | 15% | 19.81 |
| **T004** | female | 49 | Adenocarcinoma | IA | Never | (-) | (-) | <1% | 23.73 |
| **T005** | male | 63 | Adenocarcinoma | IB | Prev/curr | / | / | / | 24.09 |
| **T007** | male | 60 | Adenocarcinoma | IIB | Prev/curr | / | (-) | 90% | 18.59 |
| **T008** | male | 67 | Adenocarcinoma | IB | Prev/curr | / | (-) | 40% | 19.92 |
| **T009** | male | 42 | Adenocarcinoma | IA | Never | / | （+） | 90% | 21.61 |
| **T010** | female | 71 | Adenocarcinoma | IB | Prev/curr | / | (-) | 10% | 21.64 |

Abbreviations:Never:never smoker; Prev/Curr: previous or current smoker


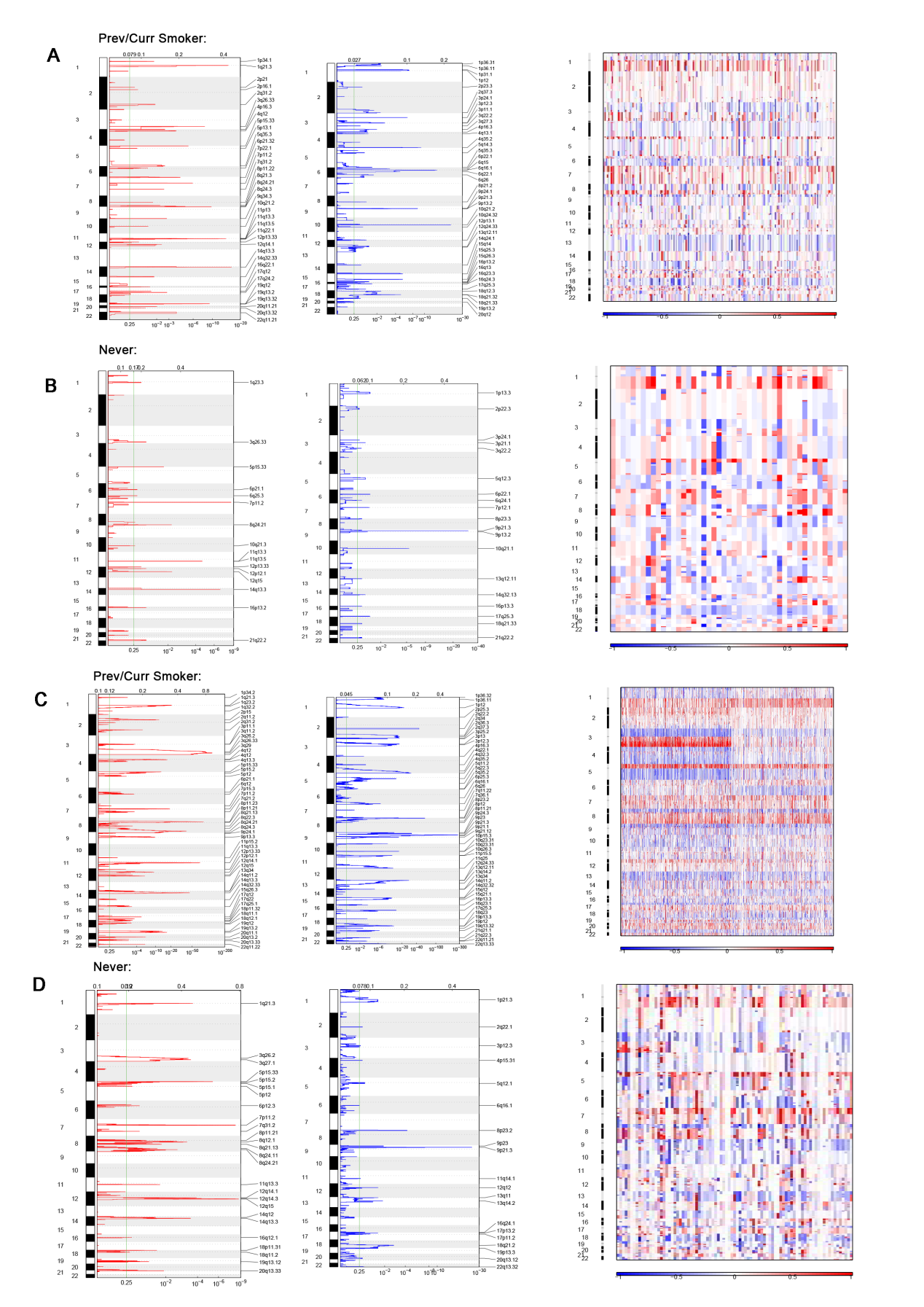


**Figure S1: Significant broad and focal copy-number alterations in the different smoking status in MSKCC dataset(A-B) and TCGA cohort(C-D)**. GISTIC2.0 identified significantly altered regions and clustering of copy number alteration data. The peaks of recurrent amplification(red) and deletion(blue) are plotted across the genome with the significantly altered chromosomes. The green lines show the significance thresholds for q values. For clustering data, Samples are arranged along the x axis and ordered according to their copy number profiles. The red-blue gradient colour shows the level of genomic alterations. According to the heatmap, the clustering of arm-level and focus CNAs can be observed clearly (colour figure online)


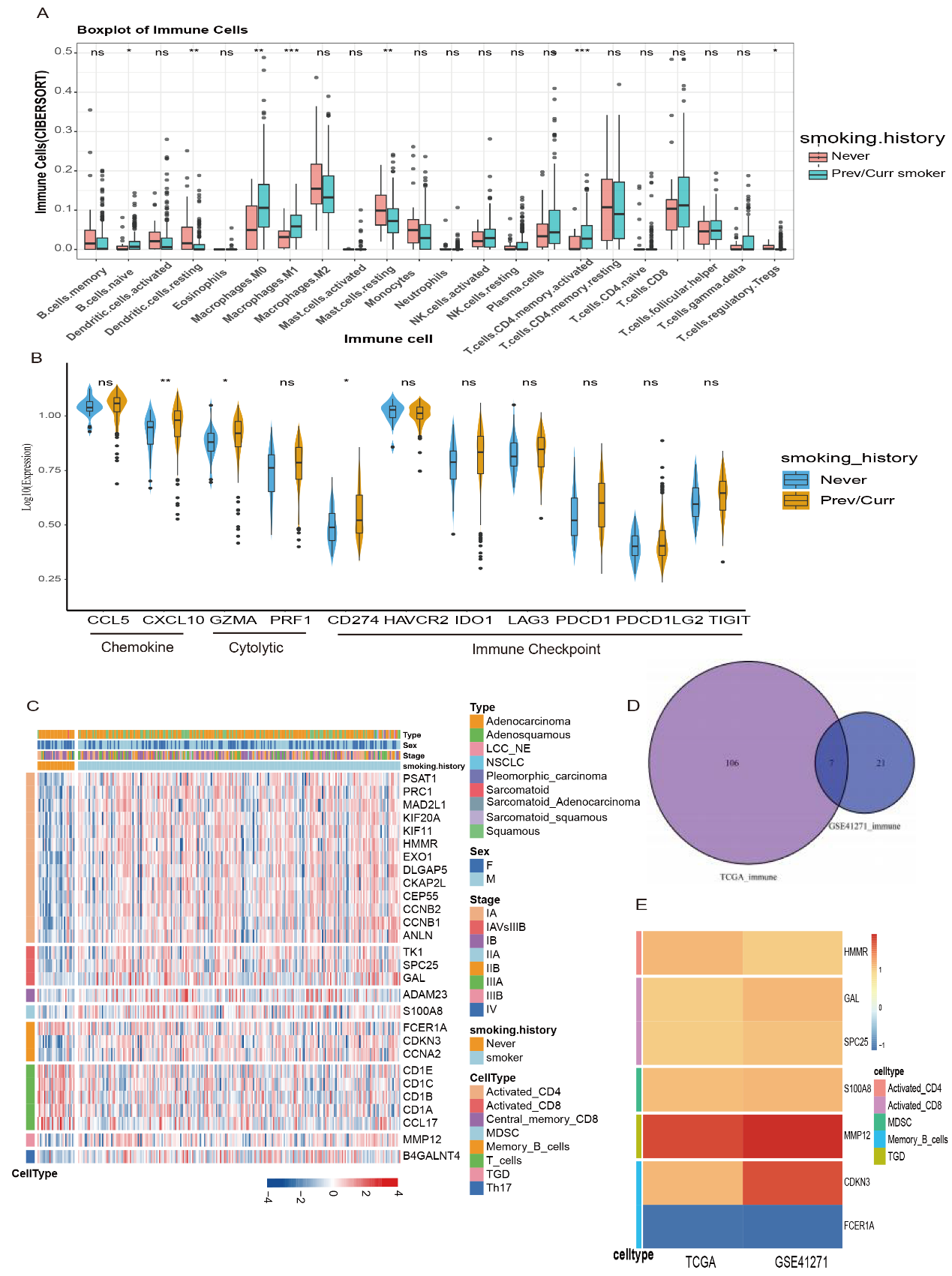


**Figure S2: Compare the immune characteristics of different smoking status in the validation cohort.**

1. CIBERSORT analyses quantifying the proportion of 22 immune cells previous/current smokers and never smokers in the NSCLC cohort from GEO cohort(GSE41271).
2. Frequencies of stimulatory immunomodulators in the different smoking status of the NSCLC cohort from GEO cohort(GSE41271).
3. Heatmap depicting 28 immune-related genes mRNA expressions with significant differences(|LogFC|≥1,P<0.05) between previous or current smokers and non-smokers in GEO validation cohort(GSE41271).The sample type , sex, stage are annotated in order in the top panel,left panel shows the types of infiltrating leukocytes marked by different genes and separated by different colors.
4. The Venn diagram shows the 7 immune-related genes shared by the NSCLC cohort from TCGA dataset and the GEO dataset that have significant differences(|LogFC|≥1, P<0.05) between different groups.

**(E)** Heatmap showing average changes in the expression levels of 7 immune-cell-related gene between the previous or current smokers and non-smokers in the TCGA and GEO dataset. The genes corresponding to the different cell types are identified by the different colors on the left side of the squares, and each square represents the logFC of a gene, filled with different back colors, from red to blue.


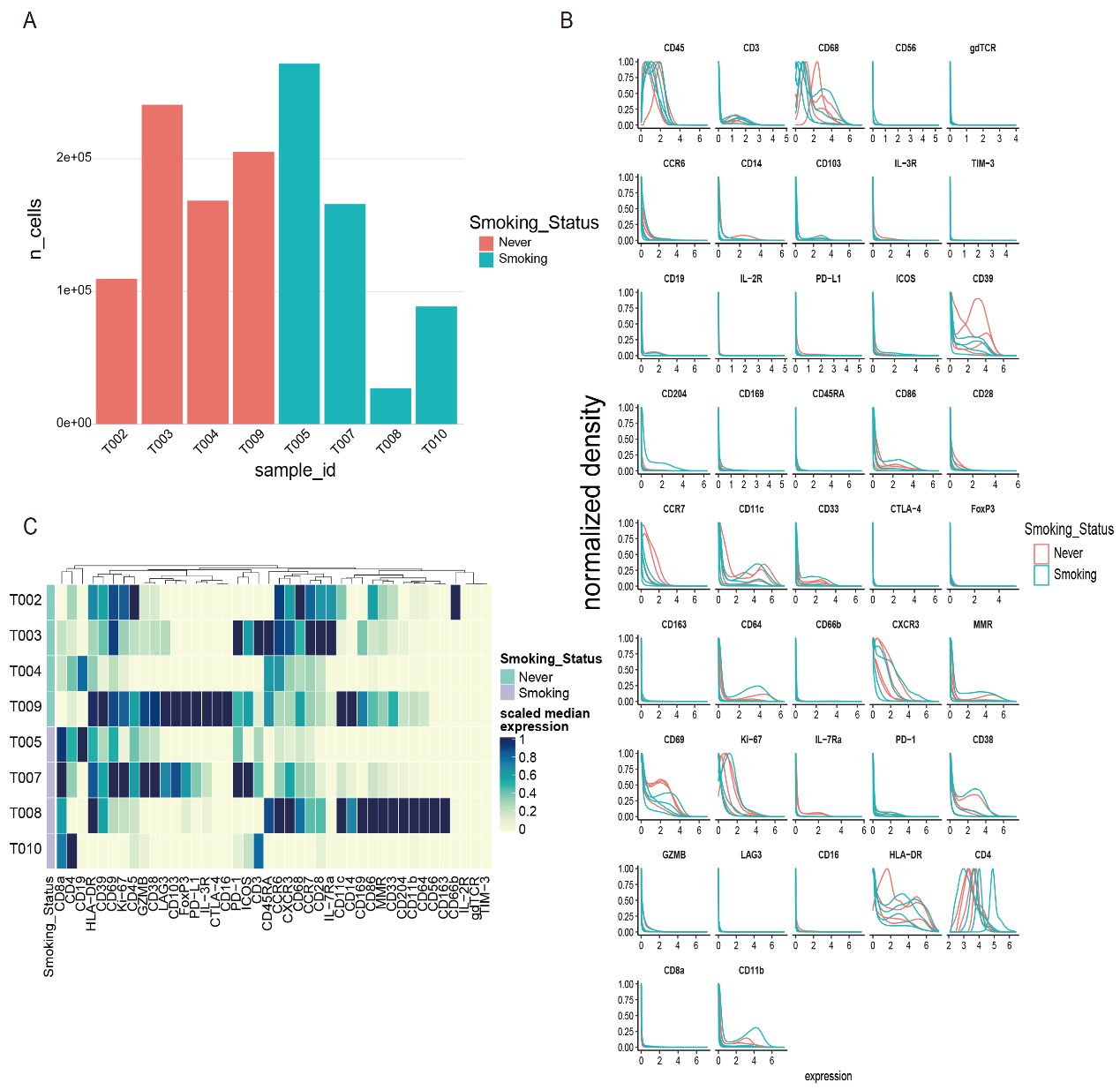


**Figure S3**：**The quality control plot of the CyTOF Dataset**.

1. Barplot showing the cell counts from each patient. Color-coded by smoking status.

**(B)**Smoothed density plot showing the expression of 42 cell surface markers. Non-smoker samples were indicated by red, while blue represents the patients with previous or current smoking history.

**(C)**Heatmap showing the median marker expression of the 42 cell surface markers across all cells grouped by each patient. Yellow codes represent lower expression while blue means higher. Row annotation bar on the left of the heatmap indicates smoker or non-smoker.


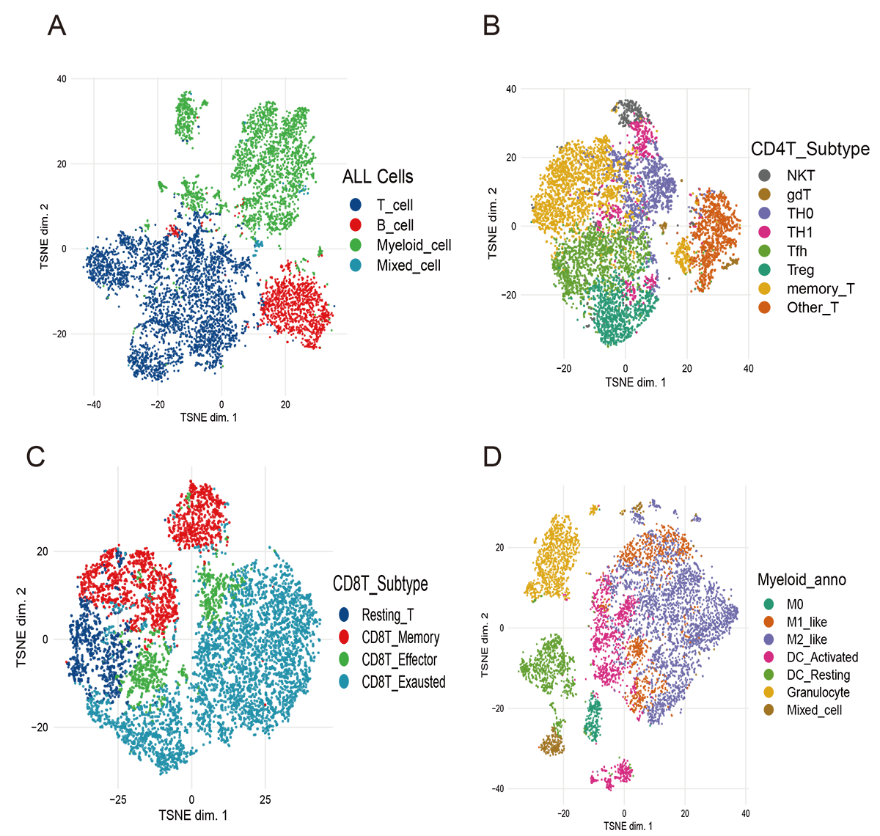


**Figure S4：Percentage of different cell populations.** t-SNE plot showing clusters of all immune cell clusters**(A)**, different CD4+ T cell clusters**(B)**, different CD8+ T cell clusters**(C)**, and different Myeloid clusters**(D)** from 8 NSCLC patients. color-coded by corresponding cluster.

**
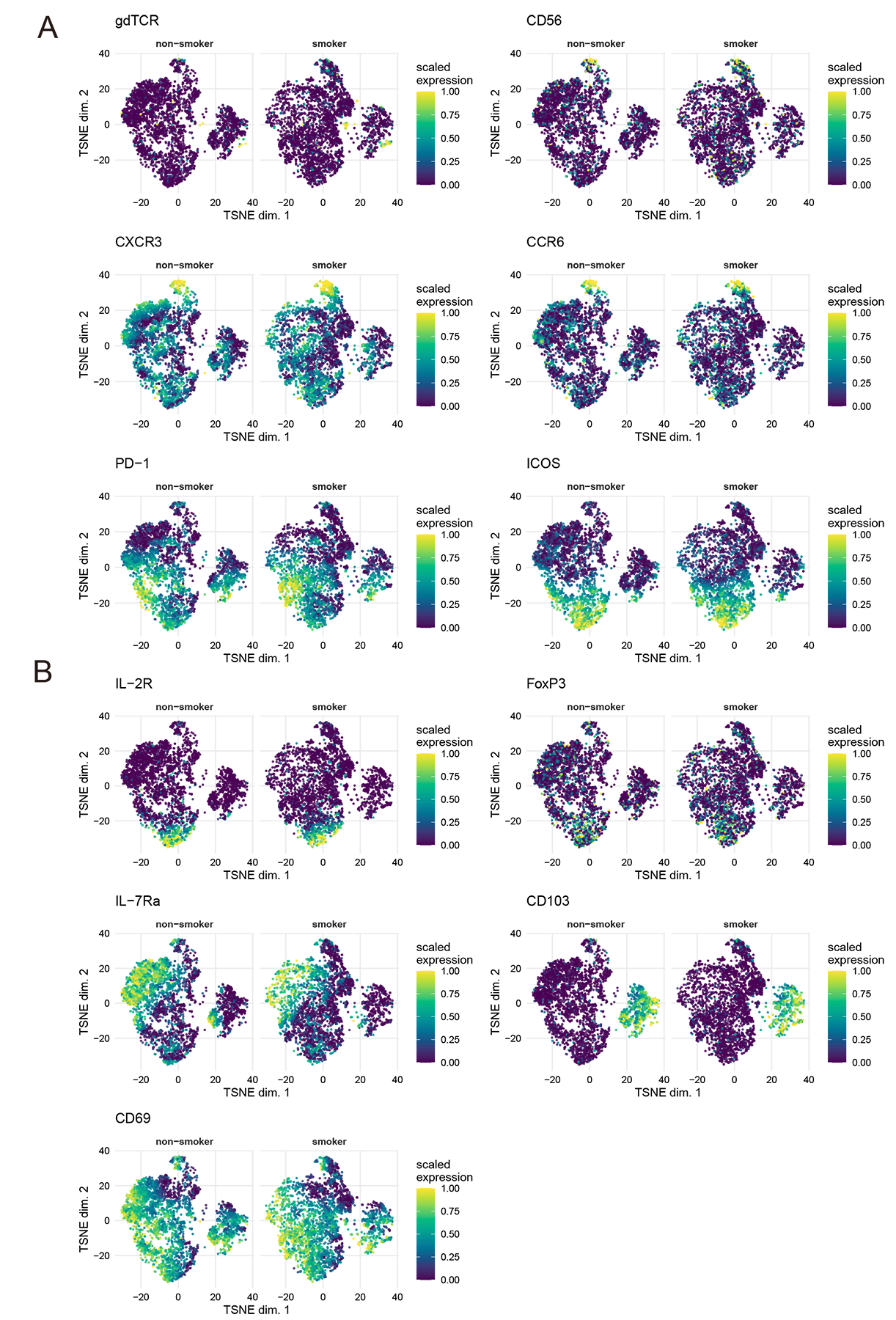
**

**Figure S5: (A-B)** t-SNE plots of markers used to annotate CD4+ T subgroups in different groups.
